# Droplet Tn-Seq combines microfluidics with Tn-Seq for identifying complex single-cell phenotypes

**DOI:** 10.1038/s41467-019-13719-9

**Published:** 2019-12-16

**Authors:** Derek Thibault, Paul A. Jensen, Stephen Wood, Christine Qabar, Stacie Clark, Mara G. Shainheit, Ralph R. Isberg, Tim van Opijnen

**Affiliations:** 1 Biology Department, Boston College, Chestnut Hill, MA 02467 USA; 2 Department of Biological Sciences, Towson University, Towson, MD 21252 USA; 3 Department of Molecular Biology and Microbiology, Tufts University School of Medicine, Boston, MA 02111 USA; 4 Present Address: Department of Bioengineering and Carl R. Woese Institute for Genomic Biology, University of Illinois at Urbana-Champaign, Urbana, IL 61801 USA

**Keywords:** Microbial genetics, Systems analysis

## Abstract

While Tn-Seq is a powerful tool to determine genome-wide bacterial fitness in high-throughput, culturing transposon-mutant libraries in pools can mask community or other complex single-cell phenotypes. Droplet Tn-Seq (dTn-Seq) solves this problem by microfluidics facilitated encapsulation of individual transposon mutants into growth medium-in-oil droplets, thereby enabling isolated growth, free from the influence of the population. Here we describe and validate microfluidic chip design, production, encapsulation, and dTn-Seq sample preparation. We determine that 1–3% of mutants in *Streptococcus pneumoniae* have a different fitness when grown in isolation and show how dTn-Seq can help identify leads for gene function, including those involved in hyper-competence, processing of alpha-1-acid glycoprotein, sensitivity against the human leukocyte elastase and microcolony formation. Additionally, we show dTn-Seq compatibility with microscopy, FACS and investigations of bacterial cell-to-cell and bacteria-host cell interactions. dTn-Seq reduces costs and retains the advantages of Tn-Seq, while expanding the method’s original applicability.

## Abstract (web summary)

Culturing transposon-mutant libraries in pools can mask complex phenotypes. Here the authors present microfluidics mediated droplet Tn-Seq, which encapsulates individual mutants, promotes isolated growth and enables cell-cell interaction analyses.

## Introduction

Transposon-insertion sequencing (Tn-Seq), and related techniques including IN-Seq, TraDIS, and HITS, have become the gold standard to determine the quantitative contribution of a gene to fitness under a specific growth condition in high-throughput and genome wide^1–4^. It has been successfully applied to bacteria, yeast, and eukaryotes^5,6^, and has enabled discoveries, including gene function, noncoding RNAs, host factors affecting disease susceptibility, bacterial transmission determinants, and vaccine targets^7–10^. One of the biggest strengths of Tn-Seq is the ability to screen hundreds of thousands of mutants in a single experiment. However, growing mutants en masse, i.e. in a pool, could mask altered fitness of certain mutants. For instance, secreted enzymes that break down complex glycans into smaller units for energy utilization can be viewed as community factors since mutants that do not produce these enzymes can “cheat” and reap the carbon-source benefits. In addition, mechanisms including frequency-dependent selection, bet-hedging, and division of labor can retain mutants with a relatively low individual fitness in a population^11,12^, which are missed by Tn-Seq. Moreover, being able to evaluate phenotypes through microscopy or FACS could create alternative opportunities to identify leads for gene function.

**Fig. 1:**
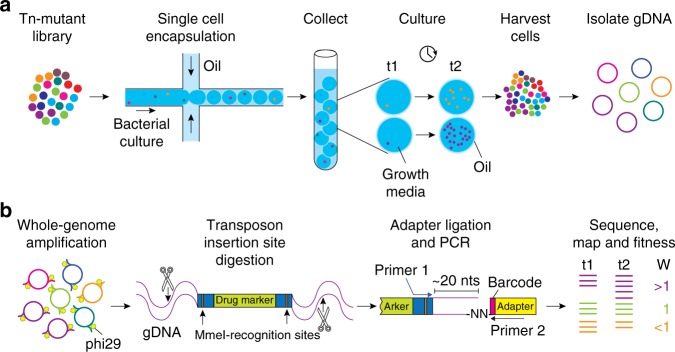
Schematic overview of droplet Tn-Seq. **a** A microfluidic device encapsulates single bacterial cells into droplets containing growth medium. Bacteria are allowed to grow within droplets, genomic DNA (gDNA) is isolated at the start of the experiment (t1) and after growth (t2). Importantly, while growth for each transposon mutant takes place in isolation, gDNA is isolated from the pooled population, enabling screening of all mutants simultaneously. **b** gDNA is then amplified with DNA polymerase phi29, digested with MmeI, an adapter is ligated, a ~180 bp fragment is produced which contains ~16 nucleotides of bacterial gDNA, defining the transposon-insertion location, followed by Illumina sequencing. Reads are demultiplexed based on the barcode in the adapter and a potential second barcode in primer 1, mapped to the genome, and fitness is calculated for each defined region.

In order to obtain a comprehensive understanding of a complex bacterial population, it is thus important to consider the growth fitness of each individual cell in isolation as well as in the context of the population. One way to address population effects that are present within a complex pool of cells, specifically a complex pool of mutants, is to create an ordered mutant library. In such a library, every bacterial mutant is separated into a different well of a microtiter plate^13–16^. Although ordered cell libraries are a tremendous resource, generating them is prohibitively expensive and time consuming. As a solution, we developed droplet Tn-Seq (dTn-Seq), by combining droplet microfluidics with Tn-Seq. In dTn-Seq, a microfluidic device enables encapsulation of millions of single bacterial mutants into micron-sized droplets, in which bacteria are cultured. Each transposon mutant thus starts off in a complex pool of mutants, is then separated and cultured in isolation, and finally cells are pooled back together. Before encapsulation and after pooling, genomic DNA is isolated for sample preparation, and the change in frequency of each mutant over the course of the experiment is determined through massively parallel sequencing to calculate the individual growth rates of each mutant^4^. Therefore, through strategic isolation and pooling, dTn-Seq enables the establishment of single-cell fitness in a genome-wide and high-throughput fashion (Fig. 1). Moreover, besides the ability to resolve complex single-cell behavior, droplets have additional advantages and applications including a substantial reduction in culture media volume (and possible expensive compounds such as host glycoproteins or proteases such as elastase), it enables analysis of bacterial microcolony formation, interbacterial interactions, and bacterial–host cell interactions through FACS and microscopy.

## Results and discussion

### Microfluidics separate single mutants into droplets

**Fig. 2:**
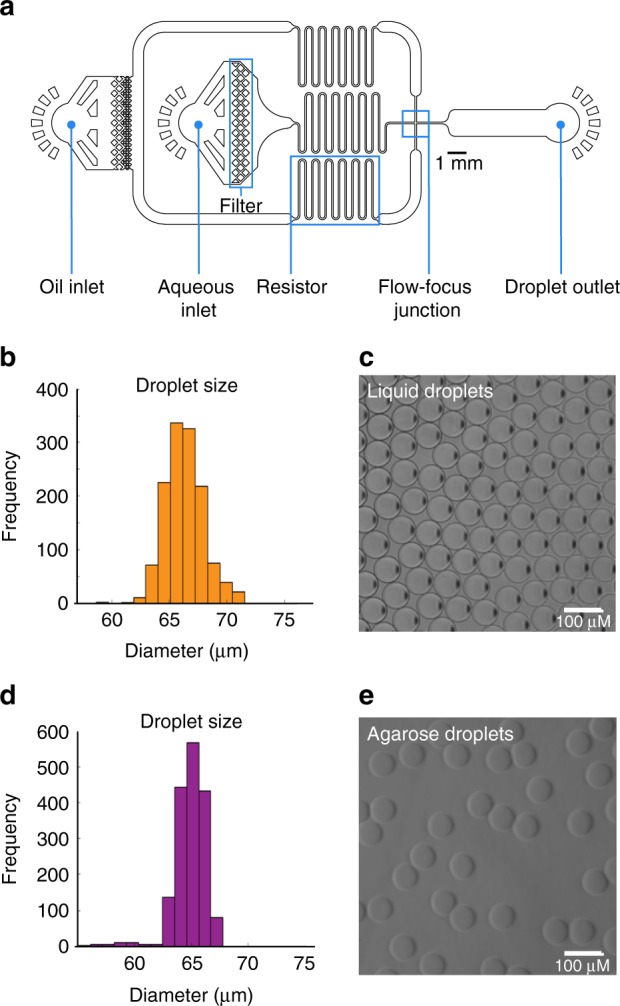
Microfluidics to generate monodisperse liquid and agarose droplets. **a** The droplet microfluidic device design allows syringe pumps to deliver surfactant in a fluorinated oil through tubing to the oil inlet and culture medium containing cells to the aqueous inlet. Filters prevent debris from clogging downstream channels while resistors reduce fluctuation in liquid flow rates. Oil separates the continuous flow of the cell culture into monodisperse droplets at the flow-focus junction. Droplets exit the device through the droplet outlet and are collected. **b**–**e** Depending on the size of the channel at the flow-focus junction, and the flow rates of the oil and aqueous phases, uniformly sized liquid or agarose droplets can be formed ensuring each encapsulated cell grows in the same volume of culture media. **b**, **d** With optimized flow rates, the average diameter of the droplets is 65–67 µm when using a 40 × 40-µm channel. **c** Liquid droplets in fluorinated oil plus surfactant. **e** Gelled agarose droplets with oil removed. Source data are available in the Source Data file.

**Fig. 3:**
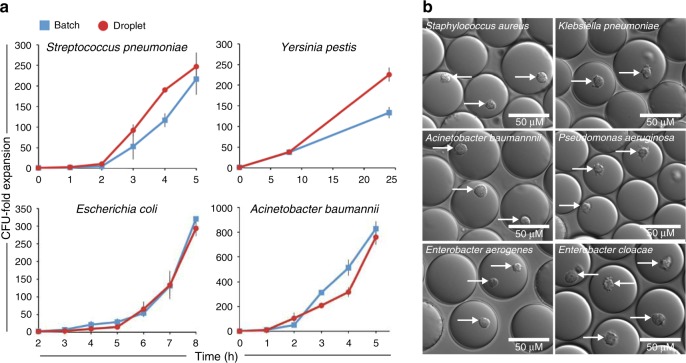
Culturing of different bacterial species in liquid and agarose droplets. **a** Both Gram-positive and -negative bacteria grow robustly in liquid droplets (red circles), and in a similar fashion to a 8 ml liquid batch culture (blue squares) (each culture was grown at least three times, error bars are standard error of the mean). **b** Agarose droplets generated by adding low melting agarose to growth medium. Agarose provides structural support and results in bacteria growing in compact microcolonies for both Gram-positive and -negative bacteria. White arrows indicate developed microcolonies. Source data are available in the Source Data file.

For “standard” Tn-Seq, complex, mutant populations are created, in which each bacterial cell contains a transposon somewhere inserted in the genome, thereby disrupting the function of the affected genetic component. To assess the fitness of each mutant in isolation from the rest of the pool and thereby possibly uncover population masking effects, a microfluidic device was designed and manufactured in-house that enables encapsulation of individual cells into growth medium-in-oil droplets, in which bacteria can be cultured (Fig. 2a; Supplementary Fig. 1, Supplementary Data 1). The size of the device channels, along with the oil and aqueous phase flow rates, determine droplet size and production frequency. For the described device this yields droplets with a ~65–67 µm diameter, ~144–157 pL volume, at a production rate of ~5 × 10^4^ droplets/min. Monodisperse droplets are composed of an outer fluorinated oil-surfactant layer and can be filled with liquid growth medium (Fig. 2b, c), which enables 5–8 generations of bacterial growth for Gram-negative and -positive bacteria alike with comparable growth dynamics to bacteria grown in liquid batch culture (e.g., an 8-ml culture; Fig. 3a). Moreover, by adding agarose to the liquid medium at a 1% concentration stable monodisperse agarose droplets are formed that provide a matrix, which supports microcolony formation (Fig. 2d, e; Fig. 3b).

### Fitness assessment of individual mutants in isolation

**Fig. 4:**
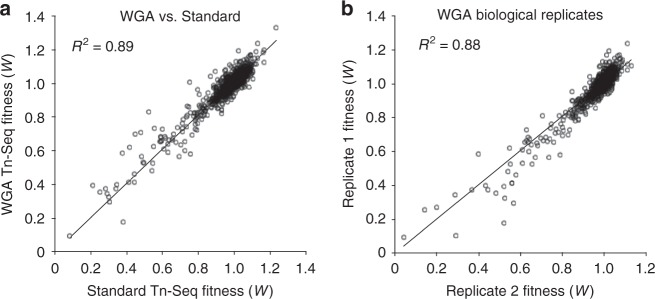
Unbiased whole-genome amplification of low-quantity genomic DNA. **a**, **b** gDNA was prepared by two different methods for transposon sequencing. For the WGA sample, 10 ng of gDNA was amplified first with DNA polymerase phi29 before MmeI digestion and adapter ligation. For the standard sample, 1 μg of gDNA was digested with MmeI, followed by adapter ligation. There is a strong correlation between fitness values obtained from WGA preparation compared with standard Tn-Seq library preparation **a**, and WGA preparation is highly reproducible **b**.

The ability to apply microfluidics to separate a pool of transposon mutants into droplets and culture them successfully creates the opportunity to assess the fitness of each mutant in isolation from the rest of the population. Similar to Tn-Seq, this can be achieved by determining the frequency of each mutant at the start and at the end of the experiment through Illumina sequencing by using the change in frequency over time to calculate each mutant’s fitness (i.e., growth rate^4,9^). Importantly, at both time points, genomic DNA (gDNA) is isolated from the entire pool of mutants; at the start of the experiment gDNA is isolated before encapsulation, while at the end of the experiment droplets are broken open using 1H, 1H, 2H, 2H-perfluoro-1-octanol (PFO), which pools bacteria back together, after which gDNA is extracted. Thus, through microfluidic device-mediated mutant separation, in-droplet culturing of bacteria and strategic pooling, droplet Tn-Seq (dTn-Seq) retains the power and genome-wide nature of Tn-Seq while enabling fitness assessment of individual mutants in isolation (Fig. 1). However, due to the droplets’ small volume, it turns out that the amount of gDNA that can be obtained from a transposon library recovered from the droplets is low (10–100 ng) and sometimes not sufficient for sample preparation. To overcome this, we introduced a whole-genome amplification (WGA) step, mediated by phi29 DNA polymerase, followed by the standard Tn-Seq steps of MmeI digestion, adapter ligation, and PCR (Fig. 1b). Optimized WGA conditions did not introduce bias into the resulting fitness data compared with the standard Tn-Seq approach (*R*^2^ = 0.89; Fig. 4a) and the reproducibility between biological replicates was high (*R*^2^ = 0.88; Fig. 4b). Alternatively, sample preparation in random barcode Tn-Seq (RB-Tn-Seq) is simpler than Tn-Seq. For those organisms for which a RB-transposon exists only a PCR on the genomic DNA is needed before sequencing^17^, making it likely that a WGA step is unnecessary if dTn-Seq is combined with RB-Tn-Seq. Lastly, dTn-Seq results could be negatively influenced if a significant proportion of droplets during encapsulation end up loaded with multiple cells. Assuming a Poisson distribution, at a cell concentration of ~2 × 10^6^ cells/ml the microfluidic device should generate a droplet population that consists of ~74% empty droplets, ~22% with single cells, and ~3% with two or more cells^18^. Encapsulation frequencies were empirically determined by mixing two *Streptococcus pneumoniae* strains, one expressing GFP and the other RFP, in a 1:1 ratio and at two different final cell concentrations of 1.75 × 10^6^ cells/ml and 2.5 × 10^6^ cells/ml. Mixed populations were encapsulated into agarose droplets, cultured, and imaged by brightfield and fluorescent microscopy to determine the cell occupation frequency. From these analyses, we determined that the device generates 78.8–83.7% empty droplets, 15.3–19.5% droplets with single cells, and ~1.0–1.8% droplets with two or more cells (Supplementary Fig. 2). These distributions are thereby within the expected range, which means that ~90% of filled droplets contain a single cell and ~10% of filled droplets will contain double-encapsulated cells. If we hypothesize that ~10% of insertions behave differently in a droplet compared with batch culture than that means that ~1% of those insertion mutants will be double encapsulated. While these sporadic double encapsulations can thereby affect a single cell’s phenotype and add noise to the fitness data, the effects should be limited. Moreover, fitness for a genetic component is, similar to Tn-Seq, calculated from multiple transposon insertions (i.e., from multiple individual mutants with a transposon inserted within the same genetic component) and for each condition multiple libraries (usually six) are sampled^4,9^. This should mostly limit the effects of double encapsulations and result in high-confidence fitness data.

### dTn-Seq uncovers population structure-dependent fitness

To determine the functionality of dTn-Seq, six transposon-insertion libraries (~10,000 mutants each) of *S. pneumoniae* were grown in batch culture as pooled populations (“standard” Tn-Seq) and encapsulated in droplets as single cells (dTn-Seq) under four different conditions: (1) growth medium with glucose as the major carbon source; (2) growth medium with the complex host glycan alpha-1-acid glycoprotein (AGP) as the major carbon source; (3) growth medium with glucose and the human leukocyte produced protease elastase, which is responsible for neutrophil-mediated killing of *S. pneumoniae*; and (4) growth medium with glucose and a 1% agarose-droplet density to assess how a solid substrate that provides structural support affects single-cell growth and promotes microcolony formation. For each gene in each condition, relative fitness was calculated as described previously, which represents the growth rate in that specific environment and enables direct cross-environment comparisons^4,9^. Fitness is thereby only considered when composed of at least three data points (transposon insertions), and significant environment effects are assessed by taking into account the amount of variance in the overall experiment and by comparing fitness through a Student’s *t* test with Bonferroni correction for multiple testing. The goal is to identify insertion mutants that have a fitness that is unique to the droplet environment of interest. For condition 1, this was achieved by comparing each gene’s fitness in the pooled batch environment with glucose as the main carbon source (the baseline environment) and the droplet environment with glucose (Supplementary Data 2). For conditions 2 and 3, comparisons were made between batch glucose and batch experiments with AGP (condition 2) or elastase (condition 3), and between droplets containing liquid growth medium with glucose and droplets, in which glucose was replaced with AGP or supplemented with elastase (Supplementary Data 3, 4). This means that a gene of interest thus shows little to no fitness effect in, for instance, batch culture with AGP, and a significant fitness effect in droplets with AGP. Lastly, for condition 4 (1% agarose), a gene of interest would have a significant fitness effect in droplets with 1% agarose compared with batch culture and droplets with liquid growth medium (Supplementary Data 2). Overall, depending on the environment, 1–3% of screened mutants from a variety of categories, including metabolism, transport, regulation, and cell wall integrity have a significantly different fitness between batch and droplet growth (Supplementary Data 2–4). This indicates that population structure, which is disrupted by separating and culturing each mutant in its own environment (i.e., a droplet), can significantly affect clonal fitness. To validate the fitness results acquired from dTn-Seq, a total of eight genes were picked from the four different conditions and explored in more detail.

### LytB affects competence and cell death in a confined space

**Fig. 5:**
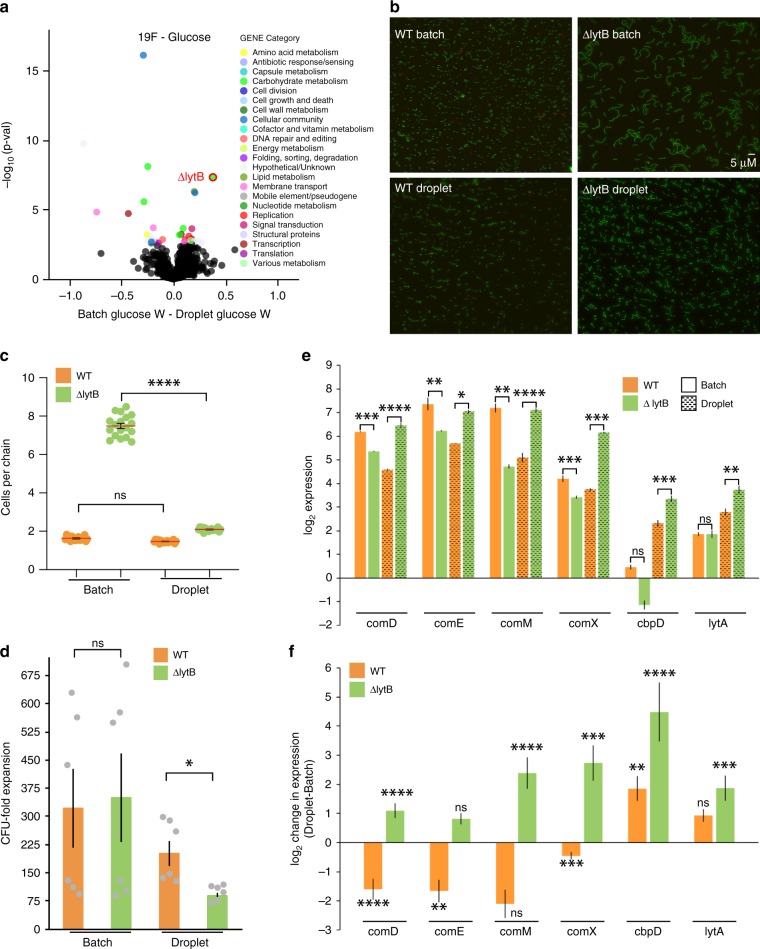
L*ytB* affects gene expression and cell death in a confined space. **a** The volcano plot shows genome-wide fitness changes from a dTn-Seq experiment between batch and droplet growth with glucose. Fitness difference is shown on the *x*-axis along with the associated *p*-value on the *y*-axis (one sample *t* test with Bonferroni correction). All significant genes are highlighted with color, which represent each gene’s functional category shown in the “Gene Category” figure key. **b** The wild-type (orange) has shorter cell-chain lengths compared with Δ*lytB* (green) in batch culture, but in droplets chain lengths are similar (**c**). **d** Loss of *lytB* causes a reduced fitness in droplets compared with batch culture. The live cell (CFU) expansion between wt and Δ*lytB* is similar in batch culture, however, in droplets Δ*lytB* grows less well then wt (*n* *=* 6; *p*-value is based on a one-way ANOVA with Bonferroni correction for multiple testing, * < 0.05). Shorter-chain lengths and less growth of Δ*lytB* in droplets could either be caused by slower growth or a higher death rate. **e** The expression of each gene relative to the control gene SPT_2222. **f** The change in expression of each gene when comparing droplet to batch. The expression of competence genes *comD*, *comE*, *comM*, and *comX* are downregulated in wt when grown in droplets, while they become upregulated in Δ*lytB*. In addition, the cell wall hydrolases *cbpD* and *lytA*, which are associated with increased cell lysis and fratricide become highly upregulated in Δ*lytB*, while only *cbpD* becomes upregulated in wt. Collectively, this suggests a role for LytB in suppressing the expression of competence-related genes in a confined environment. Each expression experiment consists of at least three biological replicates and three technical replicates each, error bars are standard error of the mean. *p*-values are based on a one-way ANOVA with Bonferroni correction for multiple testing. ns = not significant, * < 0.05, ** < 0.005, *** < 0.0005, **** < 0.0001. Source data are available in the Source Data file.

Comparing fitness data obtained from batch culture Tn-Seq with dTn-Seq in the simplest environment with glucose as the carbon source shows that a *lytB* (SPT_1238) knockout in *S. pneumoniae* strain Taiwan-19F has no effect on fitness in batch culture (batch*W*_*SPT_1238*_ = 0.98), however, when grown in droplets the mutant has a severe growth defect (droplet*W*_*SPT_1238*_ = 0.60) (Fig. 5; Supplementary Data 2). To determine whether the phenotype arises from the absence of a complex mutant population in droplets, Δ*lytB* was grown by itself in a larger 8-ml culture volume, which masked the low-fitness phenotype that was observed in droplets (Fig. 5d). It is possible that the growth difference of Δ*lytB* in a 8-ml culture compared with droplets is due to intrinsic environmental differences that could exist within the droplet. For instance, in the droplet environment, *S. pneumoniae* may experience differences in pH, CO_2_, O_2_, or H_2_O_2_ concentration, however, varying these parameters in batch culture did not affect Δ*lytB* growth (Supplementary Fig. 3). This means that the growth defect seems to be due to the small droplet environment, which suggests that in this case it is not the population structure that affects Δ*lytB* fitness, instead it is the compartment size that triggers a specific phenotype in this mutant.

LytB is part of the lytic cycle of *S. pneumoniae* and is involved in cell-chain shortening^19^. Indeed, bacterial cell chains of the Δ*lytB* mutant are increased when grown in batch. When the mutant is grown in droplets, chain lengths are shortened and similar to wild-type (wt) (Fig. 5b, c). Induction of local hypercompetence, which is in part controlled by the sensing of competence-stimulating peptide (CSP) through the ComDE two-component regulatory system, has been associated with longer cell chains^20^. Even though Δ*lytB* has shorter chains in droplets, we hypothesized that the shorter chains could be a side effect of further enhancement of competence within the confined droplet environment. Gene expression of the competence-associated genes *comD, comE, comX, comM, cbpD*, and *lytA* was determined for wt and Δ*lytB* grown in batch and in droplets. Most striking is that *comD, comE, comX*, and *comM* all have a lower expression in batch culture in the Δ*lytB* background compared with wt. However, in droplets all of these genes get downregulated in the wt, while in Δ*lytB* these genes get upregulated between ~two- and eightfold (Fig. 5e, f). In addition, in Δ*lytB* the autolysins *cbpD* and *lytA* become ~fourfold and ~32-fold upregulated, respectively, while in the wt background only *cbpD* gets upregulated (Fig. 5e, f). It is likely that due to the confined environment of the droplet, (quorum) signaling molecules, such as CSP, can build up to a high concentration, thereby triggering the competence system and induce a local hypercompetent state. While in the wt, this can be controlled and results in several competence genes becoming downregulated with no measurable effects on fitness, in the Δ*lytB* background all of the analyzed competence genes get upregulated (Fig. 5f). Especially, the increased induction of the autolysins in Δ*lytB*, which induce lysis and thus cell death, may explain why growth of the mutant in the confined droplet environment is significantly limited and cell chains are shortened. Thereby, LytB seems at least partially involved in controlling a hypercompetent phenotype from becoming too dominant through the reduction of autolysis and fratricide and thus limiting cell death. This underscores that a phenotype uncovered with approaches such as (d)Tn-Seq are not always necessarily straightforward to interpret. Because, even though we set out to identify the effect of complex communities on individual mutant fitness, the phenotype of LytB seems to be triggered by the confined environmental compartment size. Interestingly, other studies show that a buildup of signaling molecules can occur in host tissue or densely packed biofilms^21^ and affect bacterial growth states and survival^22^, which would indicate that dTn-Seq, where a similar buildup can occur, could be used to mimic and explore such environments.

### Community factor genes utilizing AGP as a carbon source

**Fig. 6:**
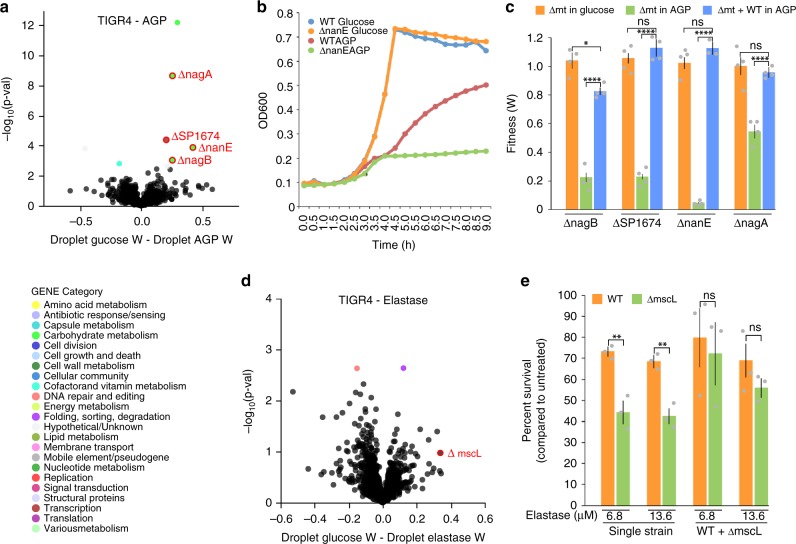
Mutants sensitive to host-specific factors can be compensated by co-culture. **a** The volcano plot shows genome-wide fitness changes from a dTn-Seq experiment comparing growth in droplets with either glucose or alpha-1-acid glycoprotein as the main carbon source. Validated genes *nagB*, SP_1674, *nanE*, and *nagA* are circled in red. **b** While Δ*nanE* (orange) grows similarly to wt (blue) in glucose, there is a growth defect for the mutant (green) compared with wt (red) when AGP is the sole carbon source. **c** Individual and mixed culturing combined with bacterial cell enumeration on agar plates containing antibiotics enabling differentiation between the wt and mt were used to determine relative fitness of the mt. While mutants grow just as well as the wt when glucose is the main carbon source (orange bars), they grow significantly slower than the wt when grown independently (green bars). However, their fitness is significantly improved when grown in the presence of the wt (blue bars), indicating that wt is providing “community support” (each growth experiment was performed at least four times, significance was determined through a one-way ANOVA with Bonferroni correction for multiple testing). **d** Volcano plot comparing growth in droplets in the absence and presence of elastase. **e** Deletion mutant Δ*mscL* (green) has reduced survival in two different concentrations of elastase compared with wt (orange) when cultured as single strains. However, Δ*mscL* survival in elastase is improved, and indistinguishable from wt, when the mutant is cultured in the presence of the wt. “Percent survival” was calculated relative to an untreated control (each experiment was performed at least three times and significance was determined through a two-way ANOVA). All error bars are standard error of the mean; ns = not significant, * < 0.05, ** < 0.005, *** < 0.0005, **** < 0.0001. Source data are available in the Source Data file.

We next tested *S. pneumoniae* libraries grown in the presence of the host factor alpha-1-acid glycoprotein (AGP). AGP has an immunomodulatory role^23^ and is also an important carbon source for many bacteria, including *S. pneumoniae*^24^. However, bacteria such as *S. pneumoniae* are unable to take up these large structures and depend on monosaccharides being liberated extracellularly by a variety of specialized secreted or cell-surface-attached bacterial enzymes^25^. Four genes (*nagB*/SP_1415, *phosphosugar-binding transcriptional regulator*/SP_1674, *nanE*/SP_1685, and *nagA*/SP_2056) were identified through dTn-Seq to have a severe growth defect in droplet culture in the presence of AGP (Fig. 6a; Supplementary Data 3). Moreover, three out of the four genes had no fitness effect in batch culture with AGP compared with the baseline environment (batch culture with glucose), while *nagA*/SP_2056 had a small defect in batch culture with AGP, but its negative fitness effect was larger in droplets with AGP (Supplementary Data 3). dTn-Seq thus indicates that each gene is dispensable when grown by itself in medium with glucose, but is important for growth when grown in isolation with AGP as the sole carbon source. These phenotypes were validated with growth curves performed with individual knockouts for each gene in growth medium with glucose or AGP as the main carbon source (Fig. 6b, c). Importantly, and as suggested by dTn-Seq, when a deletion mutant of *nagB*, SP_1674, *nanE*, or *nagA* is grown in the presence of the wild-type, i.e. in a mixed culture, the growth defect in AGP is largely masked. Even though a small defect for *nagB* and *nagA* remains (the latter not being significant), the fitness of each of these mutants is significantly improved and largely compensated by the wt (Fig. 6c). This indicates that the wild-type strain is providing community support when grown in medium with AGP as the main carbon source and can compensate the reduced fitness of the dTn-Seq-identified mutants. While none of the four genes have previously been associated with AGP in *S. pneumoniae*, or shown to be influenced by the community, each gene is associated with either regulating, releasing and/or processing AGP-linked monosaccharides. Specifically, *nagB* and *nagA* have been shown in other species to be involved in processing GlcN and GlcNAc^26^, SP_1674 is a predicted transcriptional activator of a regulon containing *nanA* and *nanB*, which have been shown to release sialic acid from complex glycan structures^25^, and *nanE* is a putative lipoprotein anchored to the membrane and important for sialic acid utilization^27^. These data reveal that dTn-Seq is indeed able to identify gene products and processes that can be shared amongst bacteria and which enable the opportunity for community resource “cheating”, which are missed with traditional Tn-Seq.

### A mechanosensitive channel protects against elastase

Other environments in which survival can possibly be influenced due to shareable resources are those in which bacteria interact with components of the host immune system. An example of this is neutrophil-mediated nonoxidative antimicrobial killing, which can involve serine proteases such as elastase, cathepsin G, and proteinase 3, which are critical for killing of a number of bacteria including *S. pneumoniae*, *Klebsiella pneumoniae*, and *Staphylococcus aureus*^28–30^. Here, bacterial growth in the absence or presence of elastase in droplet culture is compared. The use of elastase as the selective pressure introduced more variation in both the Tn-Seq and the dTn-Seq data, which resulted in the identification of only a small number of genes with a significant droplet-elastase-specific fitness effect (Supplementary Data 4). We therefore lowered our stringent cutoffs and decided to validate the large conductance mechanosensitive channel protein MscL (SP_1010). While *mscL* has one of the largest fitness defects in elastase-droplets (Fig. 6d), its fitness is composed of only four data points, and due to the larger variance in the elastase data sets this could explain why its fitness was not found to be significantly different (Fig. 6d; Supplementary Data 4). Because of the large fitness difference and the gene’s interesting annotation, we decided it would be worth exploring its phenotype. Survival experiments with wt and *ΔmscL* grown separately and in co-culture, in the presence of different concentrations of elastase confirms the negative fitness identified with dTn-Seq (Fig. 6e). Specifically, in the presence of elastase, *ΔmscL* shows an approximate 30% reduction in survival compared with the wt, while when both strains are co-cultured *ΔmscL’s* survival is indistinguishable from wt, indicating the fitness reduction due to the absence of *mscL* can be masked if at least a part of the population contains the functional gene (Fig. 6e). Since elastase is a positively charged molecule, *mscL* could be involved in creating a positively charged microenvironment thereby preventing direct access of elastase to the bacterial cell surface. *ΔmscL* mutants that are within this positively charged environment are then protected from elastase as well. This phenomenon of repelling positively charged cationic antimicrobial peptides through a positively charged membrane has been shown in bacterial species such as *S. aureus, Salmonella enterica*, and *Pseudomonas aeruginosa*^31^ (and is a phenomenon that has also been suggested to occur in *S. pneumoniae*^32,33^. Alternatively, MscL might be affecting the environment in such a manner that it leads to a reduction in activity of elastase, thereby also protecting bacteria without the functional gene. We observed that in a mixed culture the survival of the wt does become more variable, as indicated by higher variance in the data, however one could argue in favor of either hypothesis. Most importantly, this result shows that dTn-Seq is able to identify genes that interact with the host immune system, which could lead to detailed insights into how components of the immune system are involved in killing bacterial cells and how bacteria are able to avoid this.

### Capsule genes dispensable for microcolony development

**Fig. 7:**
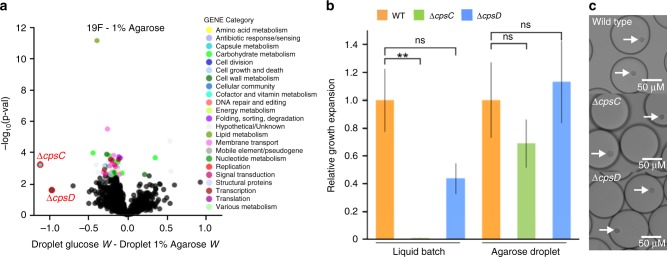
Capsule genes *cpsC* and *cpsD* are expendable in agarose droplets. **a** The volcano plot shows genome-wide fitness changes from a dTn-Seq experiment between droplet growth in glucose and droplet growth in 1% agarose. Significant genes are highlighted in color, which represent each gene’s functional category shown in the “Gene Category” figure key. **b** Wild-type (orange), Δ*cpsC* (green), and Δ*cpsD* (blue) were cultured in either liquid batch culture or agarose droplets. In each condition, the relative growth expansion was determined for each strain by counting the number of cells in the population at the beginning of the experiment and at the end after 5 h of culture. Growth expansion of the mutants are relative to the expansion of the wt strain within each growth environment (each experiment was performed at least four times). Growth is improved when the mutants are embedded in agarose (Kruskal–Wallis with Dunn’s test for multiple testing; ns = not significant, ** < 0.005, error bars are standard error of the mean). **c** Agarose droplets in oil with encapsulated *S. pneumoniae*, which have developed into compact microcolonies. Δ*cpsC* and Δ*cpsD* mutants develop similarly sized microcolonies compared to WT after 5 h. White arrows indicate microcolonies. Source data are available in the Source Data file.

We found that by adding agarose to growth medium, solid monodisperse droplets are formed that provide a stable matrix for bacteria to develop into microcolonies (Fig. 2d, e; Fig. 3b). The capsule gene *cpsC*/SPT_0394 was identified to be highly important for growth in liquid medium, but largely dispensable during microcolony development in agarose droplets (Fig. 7a). In addition, its neighboring gene *cpsD*/SPT_0395 which is in the same operon with *cpsC*, has a similar fitness profile, but while fitness for *cpsC* is significantly improved in agarose droplets, *cpsD*’s fitness just falls below the cutoff for significance (Fig. 7a), which may be due to the small number of insertions (4) in the dTn-Seq library. We decided to proceed to validate both genes to determine if these small fitness profile differences could be reflected in follow-up experiments. Indeed, when the deletion mutants are cultured in liquid medium, *ΔcpsC* hardly grows, while *ΔcpsD’s* growth is impeded, but not so much that a strong significant difference with wt is observed (Fig. 7b). Moreover, the 1% agarose-droplet environment increases fitness for both mutants and allows both to develop into robust microcolonies that are indistinguishable from wt (Fig. 7b, c). These capsule mutant growth experiments thus further confirms fitness measurements obtained by dTn-Seq and shows that some of the statistical cutoffs that we applied to reduce false positives may sometimes be slightly too conservative. Importantly, microcolony formation is central for bacterial survival in host tissue, and for instance makes bacteria such as *P. aeruginosa* less sensitive to antimicrobials^34–36^. Microcolonies and biofilms consist of clusters of bacteria, and thus dTn-Seq could provide a proxy to uncover genes that are important under such circumstances. Importantly, acapsular *S. pneumoniae* strains are often better at biofilm formation^37^, which is suggestive for the improved performance of the capsule mutants in agarose.

### Droplets enable bacterial cell and cell–host interactions

Besides the ability to separate and culture individual bacterial cells in isolation of the population, we also explored whether dTn-Seq can be used in applications to screen for phenotypes that are not directly the result of differences in growth rate. For instance, we were interested in whether interactions between bacterial cells or between bacteria and host cells can be explored, which are interactions that can critically affect bacterial population composition and/or survival^38–40^. Droplets force cells to grow in a confined space, and with the addition of agarose small spatial distances between cells can be maintained once the agarose is gelled. Agarose droplets, from which the oil has been removed, can then be easily switched between different treatments, visualized by microscopy, or sorted based on a specific fluorescently linked trait through fluorescence-activated cell sorting (FACS). Below we describe two examples in more detail that explore the potential to study cell–cell interactions utilizing agarose droplets by following: (1) competence signaling between *S. pneumoniae* strains through competence-stimulating peptide (CSP), and (2) the interaction between *Yersinia pseudotuberculosis* (*Yptb*) and bone marrow-derived macrophages (BMDMs).

**Fig. 8:**
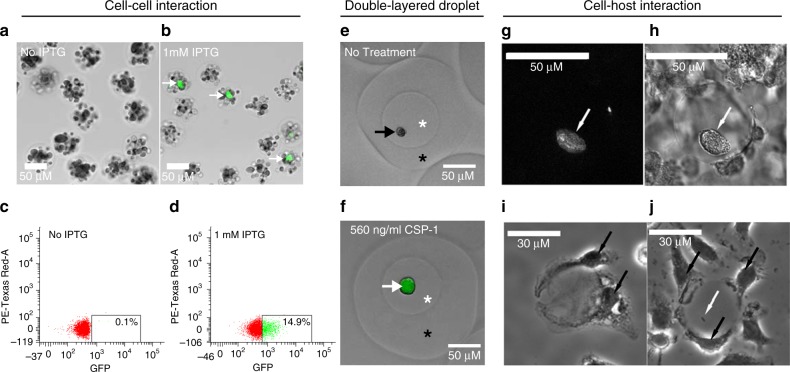
Bacterial cell–cell and cell–host interaction models. **a**–**d** *S. pneumoniae* strain sfCSPr that expresses GFP in response to CSP-1 was mixed with strain ADP112 that produces CSP-1 upon IPTG induction. A 40:1 mixture of ADP112:sfCSPr was encapsulated into agarose droplets, the oil removed, and then fluorescence and brightfield microscopy images were captured after 3 -h culture in the absence (**a**) and the presence (**b**) of 1 mM IPTG (white arrows highlight GFP-expressing sfCSPr microcolonies). Subsequent FACS analysis of agarose droplets accurately represents the predicted GFP signal frequency in the absence (**c**) and the presence (**d**) of 1 mM IPTG. **e**, **f** Gelled agarose droplets can be re-encapsulated into another droplet to form a second layer of agarose. Agarose droplets containing sfCSPr were re-encapsulated into media containing 1% agarose in the absence (**e**) or presence (**f**) of 560 ng/ml CSP-1. After 2 h of culture, the oil was removed and brightfield and fluorescence imaging revealed no GFP expression for the untreated sample, but positive GFP expression for the CSP-1 treated sample (black arrow highlights non-induced sfCSPr, white arrow highlights GFP-expressing sfCSPr, white asterisk indicates the inner droplet, black asterisk inidcates the outer droplet layer). **g**–**j** Agarose/hydrogel droplets containing *Yersinia pseudotuberculosis* (*Yptb*) were exposed to murine bone marrow-derived macrophages (BMDMs). *Yptb* strain IP2666 GFP + was grown in droplets overnight and visualized by fluorescence (**g**) and brightfield (**h**) microscopy. After a 1-h incubation, BMDMs can attach to oil-free empty droplets (**i**) or droplets containing *Yptb* cells (**j**) (white arrows indicate *Yptb* cells and black arrows indicate BMDMs).

CSP signaling in *S. pneumoniae* is part of a quorum-sensing process used to trigger competence mechanisms including uptake and recombination of the extracellular DNA, and can either induce or protect from fratricide, depending on the competence state of the cell^41–43^. We generated the *S. pneumoniae* strain sfCSPr (Supplementary Data 5, 6), by fusing the CSP promoter (SPD_2065/*comC*) with the superfolder green-fluorescent protein (sfGFP)^44^. sfCSPr thereby produces a GFP signal in the presence of CSP-1 (*comC*). sfCSPr was mixed with *S. pneumoniae* strain ADP112, a strain that produces CSP upon addition of isopropyl-β-D-thiogalactopyranoside (IPTG)^45^ at a ratio of 1:40 (sfCSPr:ADP112). The cell mixture was encapsulated at a concentration of ~7 × 10^7^ cells/ml into agarose droplets, which, following the Poisson distribution along with our own empirical measurements (Supplementary Fig. 2), means that ~16% of droplets should contain a single sfCSPr cell, while all droplets should contain at least one bacterial ADP112 cell. Robust microcolonies were allowed to develop for 3 h in the absence or presence of 1 mM IPTG, after which droplets were analyzed by brightfield and fluorescent microscopy and FACS. As expected, no GFP signal could be observed in the absence of IPTG either by fluorescent microscopy or FACS (Fig. 8a, c), while a robust GFP signal could be observed in droplets that were incubated with IPTG (Fig. 8b, d). Moreover, FACS was used to determine that ~15% of droplets contained a GFP signal, which is similar to the expected percentage (Fig. 8d; Supplementary Fig. 4). Lastly, we found that after removing the oil from an agarose droplet, it can be re-encapsualted into a second droplet, thereby creating a second droplet layer. To explore this droplet model, sfCSPr was encapsulated into an agarose droplet and a microcolony was allowed to develop. Subsequently, the oil was removed with PFO after which the gelled agarose droplet was re-encapsulated on a device with slightly bigger channels (80 × 40 μm) and no filter downstream of the aqueous inlet, in the presence of CSP, medium, and agarose. Within 2 h, CSP had diffused from the second layer into the first layer and reached the microcolony, as demonstrated by induction of a strong GFP signal (Fig. 8e, f). This shows that quorum signals such as CSP (or for that matter probably any diffusible compound) can freely diffuse throughout an agarose droplet, enabling the possibility to screen for diffusible signal-mediated interactions between bacteria, or for instance compounds that inhibit or stimulate growth, which in combination with FACS could be turned into a high-throughput screen.

To follow interactions between bacteria and host cells, agarose droplets were combined with *Yersinia pseudotuberculosis* and bone marrow-derived macrophages (BMDMs). Extracellular bacterial pathogens, such as *Y. pseudotuberculosis (Yptb)*, can establish microcolonies in deep tissue sites, such as the liver and spleen, and then break free from the colonies to establish new centers of replication^46^. These extracellular clusters become surrounded by host innate immune cells, which include immune cells such as macrophages and inflammatory monocytes. Macrophages, however, do not come in direct contact with the microcolonies, but the bacteria are exposed to extracellularly secreted factors produced by macrophages. To mimic this structure in vitro, *Yptb* was encapsulated into agarose/hydrogel droplets, which were able to support *Yptb* growth as clusters (Fig. 8g, h). BMDMs from C57/BL6 mice were added to the agarose droplets after the establishment of the *Yptb* microcolony, which resulted in a topology of microcolony-immune cell composition that mimics what is observed in the mouse spleen^47^. The adhesion of macrophages does not require the productive replication of bacteria or the establishment of a mature microcolony, as droplets lacking *Yptb* (Fig. 8i), or having only a single bacterium (Fig. 8j) also supported the adhesion of BMDMs to agarose droplets containing hydrogel. This setup could thereby enable screening for non-contact-mediated interactions between *Yersinia* and macrophages, which are known to affect virulence and clearance of the bacterium^47^.

### dTn-Seq as a valuable complementary tool to Tn-Seq

In order to improve our understanding of how pathogenic bacteria cause disease or evolve antibiotic resistance, it is critical to develop approaches to determine how bacteria deal with or adapt to environmental stress, such as the host immune system, varying carbon sources, and antibiotics. In the past 10 years, the ease of use and high-throughput nature of Tn-Seq methods, such as Tn-Seq, IN-Seq, TraDIS, and HITS, have allowed for faster and easier strategies to obtain leads for gene function and increased insight into how bacteria cause disease. However, we hypothesized that these methods may fail to identify certain phenotypes, such as those caused by population effects within batch culture of the complex transposon libraries. Therefore, by combining Tn-Seq with droplet microfluidics, we developed dTn-Seq, a method which efficiently separates and cultures transposon mutants in their own compartment (a droplet), and enables identification of mutants whose fitness is affected by the presence of other (mutant) strains in the population. To illustrate the validity of dTn-Seq, we describe: (1) a novel role for *lytB* which contributes to controlling local hypercompetence and limiting cell death; (2) four genes (*nagB*, *phosphosugar-binding transcriptional regulator*, *nanE*, and *nagA*) that are involved in the community usage of host alpha-1-acid glycoprotein as an energy source; (3) an ion channel (*mscL*) that improves survival in the presence of neutrophil elastase; and (4) two capsule genes (*cpsC* and *cpsD*) that are necessary for planktonic culture, but are dispensable during microcolony formation. Moreover, we show that the droplet environment enables robust growth of Gram-positive and -negative species alike providing support that dTn-Seq can be implemented for any species for which Tn-Seq exists. We show that droplets can be imaged, that agarose droplets are sortable via FACS, and how dTn-Seq could be implemented to study phenotypes such as bacterial cell–cell and/or bacteria–host cell interactions.

Lastly, the droplet environment also potentially reduces the amount of compounds and chemicals needed to perform an experiment. For instance, elastase and AGP used in the experiments described above are expensive compounds, and can quickly increase experimental costs. While we normally perform Tn-Seq in ~8-ml cultures, an equivalent number of mutants can be screened with dTn-Seq in 400 μl, allowing for a ~20× reduction in volume and cost. While we recognize that syringe pumps (~$500) and microfluidic mold fabrication and chip production (~$200) have some start-up costs, these costs are quickly off-set because: (1) a single mold can be used to generate many PDMS chips; (2) each PDMS chip contains 38 separate devices; and (3) each device can be re-used. Moreover, a more standard method to determine single-mutant phenotypes is through the use of an ordered array. While the construction of such an array is costly and time consuming, utilizing an array to perform experiments is also expensive, and would require even more material than a standard Tn-Seq, let alone dTn-Seq. For instance, screening ~1500 *S. pneumoniae* nonessential genes with an ordered mutant library in a 96-well format, requires a total culture volume of at least 225 ml (assuming ~150 μl per well). Thus, the significant reduction in volume, along with the inherent difficulty of generating and utilizing an ordered mutant library makes dTn-Seq an attractive alternative, or at the very least, complementary.

In conclusion, dTn-Seq is applicable to a wide variety of bacteria and can be used as an extension to any variation of Tn-Seq to uncover (complex) single-cell phenotypes due to population affects, environment size, or interactions with the extracellular microbial and/or host environment.

## Methods

### Bacterial strains, growth, and media

Sequencing and validation experiments were performed using *Streptococcus pneumoniae* strains TIGR4 (NCBI Reference Sequence: NC_003028.3), and Taiwan-19F (NC_012469.1). Other species used in the study for growth models were *Yersinia pseudotuberculosis* (*IP2666*), *Yersinia pestis* (KIM6, pCD1-negative and pgm-negative), *Escherichia coli* (DH5-α), *Acinetobacter baumannii* (ATCC 17978), *Staphylococcus aureus* (RN1, NR-45904), *Klebsiella pneumoniae* (UHKPC57, NR-44357), *Pseudomonas aeruginosa* (PA14), *Enterobacter aerogenes* (NRRL B-115), and *Enterobacter cloacae* (NRRL B-412). Except for specific growth and selection experiments, the *S. pneumoniae* strains were cultured either statically in Todd Hewitt broth supplemented with yeast extract (THY) plus 5 µl/ml Oxyrase (Oxyrase, Inc.) and 150 U/ml catalase (Worthington Bio Corp LS001896), or on Sheep’s blood agar plates at 37 °C in a 5% CO_2_ atmosphere. *Y. pseudotuberculosis* was cultured in 2xYT, and *Y. pestis* was cultured in brain heart infusion media or on blood agar while all other strains were cultured in Luria-Bertani (LB) broth or on LB agar at 37 °C. Unless otherwise noted, cells were cultured to the exponential phase before being washed in PBS and diluted down into the appropriate media. Growth curves were generated using a Tecan Infinite 200 PRO plate reader.

### Microfluidic device production

Microfluidic device masks were designed using AutoCad 2016 software (AutoDesk) (Supplementary File 1), and photomasks made of acetate transparency film were ordered from CAD/Art Services, Inc. (Bandon, OR). The silicon mold and final microfluidic chip fabrication were performed at the Integrated Sciences Cleanroom and Nanofabrication Facility at Boston College. A master mold was fabricated by coating a silicon wafer with the negative photoresist SU-8 3025 (MicroChem) using a spin coater (Laurell) to generate SU-8 with a thickness of 40 μm, and set by baking at 95 °C. The photomask was aligned with the silicon wafer and UV exposed followed by a post-exposure bake ramping from 65 °C to 95 °C over 4 min. The mold was developed using SU-8 developer (MicroChem) per the manufacturer's guidelines and rinsed with isopropanol and dH_2_O followed by heating from 100 °C to 200 °C over 5 min. The polydimethylsiloxane **(**PDMS) chip was generated by mixing PDMS and curing agent (Dow Corning, Sylgard 184) in a 10:1 ratio and added to the mold, degassed with a vacuum, and polymerized at 65 °C overnight. Polymerized PDMS was cut from the mold, and a biopsy punch (0.75 mm—Shoney Scientific) was used on top of a self-healing cutting mat to create ports for tubing (PE-2 tubing—Intramedic) just below the three star patterns for each device (Fig. 2a). PDMS slabs were bonded to glass (Corning −2947, 75 × 50 mm) at a clean room by first washing the glass with acetone and isopropanol in a sonicator bath while the PDMS was washed with isopropanol, followed by thorough drying with filtered nitrogen gas. The channel side of the PDMS slab and the glass slide were treated with plasma (400 sccm flow; 400 watts; 45 s) using a faraday barrel screen. Plasma-treated surfaces were quickly brought into contact and pressed together and then placed at 65 °C for 10 min to complete bonding.

### Droplet production and culturing in droplets

Before droplet production, the device’s aqueous channel was primed with Aquapel (Aquapel #47100) and then flushed with fluorinated oil (Novec 7500 oil; 3 M #98-0212-2928-5) (Fig. 2a). Devices were used immediately or incubated overnight at 65 °C, covered in scotch tape, and then stored in the dark for several weeks before use. To produce droplets, a 1-ml syringe (BD, 309628) was filled with 1.5% of PicoSurf-1 in Novec 7500 oil (Sphere Fluidics, C024) while another 1-ml syringe was filled with thoroughly vortexed cell culture, and then both were hooked into syringe pumps (Cole-Parmer Instrument Co., 00280QP). PE-2 tubing was used to transfer PicoSurf-1 oil to the “oil inlet”, cell culture to the “aqueous inlet”, and collect droplets from the “droplet outlet” (Fig. 2a). A syringe pump rate of 500 μl/h was used for both oil and aqueous phases, and encapsulation and collection were performed for 30 min. The entire droplet production system can be seen in Supplementary Fig. 1. To generate agarose droplets, the entire droplet production system was placed in a 37 °C warm room. 1% Seaplaque agarose (Lonza – 50101) was added to growth media and then heated until dissolved. The liquid agarose was then filtered (0.45 μm) after which thoroughly vortexed cells were added to the 37 °C pre-warmed agarose solution. Encapsulation was then performed the same as for liquid droplets described above. After production, agarose droplets were gelled at 4 °C for 12 min with occasional shaking, and then cultured. Single-droplet devices may be re-used for either liquid or agarose droplets if the entire device is flushed immediately after use with Novec 7500 oil (no surfactant) followed by clearing the device with air using an empty syringe. Scotch tape is then used to cover the ports of all devices to prevent debris from entering and clogging the channels. To determine the monodispersity of liquid or agarose droplets, first microscopic images were taken and then processed in ImageJ^48^ to measure the range of droplet diameters. To generate growth curves for liquid or agarose-droplet culture, a fraction of the droplet culture was collected and quickly broken open with a final working concentration of 16% 1H, 1H, 2H, 2H-perfluoro-1-octanol (PFO; Sigma-Aldrich, 370533), which separates oil and aqueous phases. For liquid droplets, the upper aqueous culture phase was immediately plated for live cell counts. For agarose droplets, the upper aqueous phase was added to a dounce homogenizer to break up the agarose and release cells for live cell plating. Live cell expansion was calculated by dividing live cell counts (CFU) at every time point by the CFU count at the beginning of each experiment.

### Frequency of cell-doublet encapsulation in droplets

In order to generate droplets that contained single bacteria, the cell culture was first diluted based on droplet size according to a Poisson distribution^18^. Frequency of cell-doublets in droplets was then determined by first mixing GFP-fluorescing (JWV500^44^) and RFP-fluorescing (MK119^49^) *Streptococcus pneumoniae* strains in an equal ratio, at two different final cell concentrations (1.75 × 10^6^ cell/ml and 2.5 × 10^6^ cell/ml), encapsulating and growing in 1% agarose droplets, and finally using brightfield and fluorescence imaging to determine encapsulation frequencies. Specifically, CellProfiler software^50^ was used to automatically identify individual droplets as well as red or green fluorescence. If a red or green microcolony resided within 15 pixels of droplet area, it was considered a part of that droplet. Counts of the red, green, red/green, and total number of droplets were then used to assess encapsulation frequencies. Measurements were an average of seven images with at least 10,000 total droplets counted for each of the two cell concentrations.

### Visualization of cells and droplets

Images of cells and droplets were captured with an Olympus IX83 inverted microscope. For planktonic batch culture, 10 μl of cells were stained with 0.5 μl green-fluorescent SYTO-9 (1:10 dilution in PBS) and 0.5 μl red-fluorescent propidium iodide (1:10 dilution in PBS) (Thermo Fisher Scientific, L34856). Batch culture cells were then mounted between an agar pad and coverslip for visualization. All droplet images were produced by mounting samples on a glass slide with coverslip spacers to prevent droplets from being compressed.

### Transposon library construction and selection experiments

Library construction using the mariner transposon Magellan6 was performed as previously described^4,9,51^. The transposon lacks transcriptional terminators allowing for read-through transcription, and additionally has stop codons in all three frames in either orientation to prevent aberrant translational products. Six independent transposon libraries were produced and utilized for each experimental condition using *Streptococcus pneumoniae* strains TIGR4 or Taiwan-19F. Each transposon library consists of at least 10,000 total mutants. The environmental conditions for selection experiments included: (1) growth in semi-defined minimal media (SDMM)^51^ at pH 7.3 supplemented with either 20 mM glucose or 5 μM human alpha-1-acid glycoprotein (Sigma, G9885) as a carbon source, (2) 5 μM purified neutrophil elastase as a host immune stress (Elastin Products Company, Inc, SE563), or (3) 1% agarose as an embeddable substrate (Lonza, Seaplaque, 50101). Every selection experiment was cultured statically at 37 °C in a 5% CO_2_ atmosphere.

### Sample preparation, sequencing, and fitness calculations

Genomic DNA (gDNA) was extracted from *S. pneumoniae* using the DNeasy Blood & Tissue Kit according to the manufacturer’s guidelines for Gram-positive bacteria (Qiagen, 69506). DNA adaptor barcodes were made by mixing an equal volume of primers ADBC-F and ADBC-R (Supplementary Data 5) at a concentration of 0.2 nM in buffer (10 mM Tris-base, 50 mM NaCl, 1 mM EDTA, pH 8), followed by incubation at 95 °C for 3 min, 60 °C 10 min, 55 °C 10 min, 50 °C 20 min, 45 °C 30 min, 42.5 °C 15 min, 40 °C 15 min, 21 °C 1 min, and held at 4 °C. Illumina DNA sample preparation for Tn-Seq was performed depending on the amount of gDNA collected. High gDNA amounts (> 1 μg) were prepared with a standard Illumina preparation method for Tn-Seq^4,52^. Low gDNA input amounts (10–100 ng) were prepared by first performing whole-genome amplification (WGA) on the gDNA sample using phi29 DNA polymerase (NEB - M0269S). The dTn-Seq sample preparation was performed as follows: (1) 10 ng of gDNA was mixed with 10 μM exo-resistant primer (MCLAB – ERRP-100), 2.5 mM dNTP, and 1X phi29 DNA polymerase reaction buffer, in a total volume of 26.25 μl, and incubated at 95 °C for 3 min and then placed on ice. Next 1× BSA (NEB), and two units of phi29 (0.57 U/ml), were added to the reaction, and incubated for 7 h at 30 °C, 10 min at 65 °C, and held at 4 °C. (2) In total, 10 μl of magnetic beads (Axygen – AxyPrep Mag PCR Clean-up Kit, MAGPCRCL50) were mixed with 30 μl of freshly made polyethylene glycol (PEG) solution (20% PEG 8000, 2.5 M NaCl, 10 mM Tris-base, 1 mM EDTA, 0.05% tween20, pH 8) and added to the 30 μl of sample, mixed, and incubated at room temperature for 20 min. A magnet was used to separate the bead/DNA complex from the PEG solution, and the beads were washed three times in 200 μl 70% ethanol (all magnetic bead washes were performed this way). Beads were then dried for 3 min at room temperature, and DNA was eluted off the beads with 12.7 μl of dH_2_O. (3) In all, 11.49 μl of phi29 amplified DNA was then added to a MmeI digestion mix (two units NEB MmeI enzyme, 50 μM SAM, 1× CutSmart Buffer) in a total volume of 20 μl, and incubated for 2.5 h at 37 °C followed by 20 min at 65 °C. (4) In all, 1 μl of alkaline phosphatase (NEB - M0290S Calf Intestinal, CIP) was added to the sample and incubated for 1 h at 37 °C. (5) In total, 10 μl of magnetic beads plus 20 μl PEG solution per sample were used to wash the sample followed by elution in 14.3 μl of dH_2_O. (6) T4 DNA ligase (NEB M0202L) was used to ligate DNA adapter barcodes by adding 13.12 μl DNA to 1 μl of 1:5 diluted adapter, 1× T4 DNA Ligase Reaction Buffer, and 400 units T4 DNA ligase, followed by incubation at 16 °C for 16 h, 65 °C for 10 min, and held at 10 °C. (7) In all, 10 μl magnetic beads plus 20 μl PEG solution were used to wash the sample followed by elution in 36 μl of dH_2_O. (8) Adapter ligated DNA was then PCR amplified using Q5 high-fidelity DNA polymerase (NEB – M0491L) by adding 34 μl of DNA to 1X Q5 reaction buffer, 10 mM dNTPs, 0.45 μM of each primer (P1-M6-GAT-MmeI; P2-ADPT-Tnseq-primer; Supplementary Data 5), one unit Q5 DNA polymerase, and incubated at 98 °C for 30 s, and 18–22 cycles of 98 °C for 10 s, 62 °C for 30 s, 72 °C for 15 s, followed by 72 °C for 2 min, and a 10 °C hold. (9) PCR products were gel purified and sequenced on an Illumina NextSeq 500 according to the manufacturer's protocol. Sequence analysis was performed with a series of in-house scripts^4,53,54^. The fitness of a single mutant (*W*_*i*_) is calculated by comparing the fold expansion of the mutant to the fold expansion of the population and is determined by the following equation^4,55^:

$$W_i = \frac{{\ln \left( {N_i\left( {t_2} \right) \times d/N_i\left( {t_1} \right)} \right)}}{{{\mathrm{ln}}((1 - N_i\left( {t_2} \right)) \times d/(1 - N_i\left( {t_1} \right)))}},$$

in which *N*_*i*_*(t*_*1*_*)* and *N*_*i*_*(t*_*2*_*)* are the mutant frequency at the beginning and end of the experiment, respectively, and *d* is the population expansion. The final average fitness, standard deviation, and standard error are calculated across all insertions within a gene, and since fitness is calculated using the expansion factor of the population, *W*_*i*_ becomes independent of time, therefore allowing comparisons between different strains and conditions across different experiments. To determine whether fitness effects are significantly different between conditions, three requirements had to be fulfilled: (1) *W*_*i*_ is calculated from at least three data points, (2) the difference in fitness between conditions has to be larger than 10% (thus *W*_*i*_  − *W*_*j*_ = < −0.10 or > 0.10), and (3) the difference in fitness has to be significantly different in a one sample *t* test with Bonferroni correction for multiple testing. Fitness volcano plots were generated using Vega-Lite^56^.

### Mutant generation

Gene knockouts were constructed by replacing the entire coding sequence with a chloramphenicol or spectinomycin resistance cassette through overlap extension PCR. Construction of PCR products for gene replacement and transformation of *Streptococcus pneumoniae* were performed as described previously^4,57^. Generated mutant strains and primers for marked deletions can be found in Supplementary Information (Supplementary Data 5, 6).

### Co-culture assays

To validate genetic phenotypes associated with carbon utilization from AGP or stress from neutrophil elastase, single-gene mutants (mt) were co-cultured with their wild-type parental strain (wt) in a 1:20 ratio (mt:wt). Mutant and wt frequencies were calculated by live cell plating on blood agar plates with or without antibiotic selection for the mutant. Overall growth of the mutant is then represented either as fitness, which reflects the growth rate and is described above, or as percent survival which is calculated relative to the untreated control.

### Gene expression analysis

Immediately after culture *Streptococcus pneumoniae* were pelleted and snap-frozen in an ethanol/dry-ice bath, followed by RNA isolation using RNeasy Mini Kit (Qiagen, 74106) according to the manufacturer’s guidelines. RNA was treated to remove genomic DNA with TURBO DNA-free kit (Invitrogen, AM1907). cDNA was made from 400 ng of DNA-free RNA using iScript Reverse Transcription Supermix (Bio-Rad, 1708841). Primers for quantitative real-time PCR (qRT-PCR) were designed using Primer3 software^58,59^ (Supplementary Data 5). qRT-PCR was performed with iTaq SYBR Green Supermix (Bio-Rad, 1725124) using 2 μl of cDNA in a MyiQ Real-Time PCR Detection System (Bio-Rad). Each sample was measured in three technical and biological triplicates and normalized to the 50S ribosomal gene SPT_2222 (*rplI*).

### Bacterial cell–cell and cell–host interactions in droplets

For the cell–cell interaction model, we produced a *Streptococcus pneumoniae* strain, called sfCSPr, which generates a GFP signal upon treatment with CSP-1 (SPD_2065/*comC*) (Supplementary Data 5, 6). This was accomplished by fusing the promoter region of *comC* in front of a coding sequence that produces superfolder green-fluorescent protein (GFP)^44^, along with an antibiotic selection marker, and integrated into the genome locus 1902556-1902558 of *S. pneumoniae* strain D39. The cell–cell interaction was then set up by mixing the sfCSPr with ADP112, a D39 strain which produces CSP upon treatment with isopropyl-β-D-thiogalactopyranoside (IPTG)^45^ in a 40:1 mixture (ADP112:sfCSPr). We chose this ratio to guarantee that enough CSP would be produced by ADP112 to trigger a strong GFP signal in sfCSPr. Cell cultures were first grown in C + Y media at pH 6.8. Next, the cell mixture was encapsulated into 1% agarose droplets, and the oil removed with a final concentration of 16% 1H, 1H, 2H, 2H-perfluoro-1-octanol (PFO). Next, agarose droplets were washed several times in C + Y media, cultured in C + Y media at pH 7.6 in the absence and the presence of 1 mM IPTG for 3 h, and then finally visualized with brightfield and fluorescence microscopy. For the cell–host interaction model, droplets were made with 1% ultralow melt agarose containing 25% hydrogel (ESI BIO). After oil removal with PFO, the droplet-embedded *Yptb* cells (IP2666 or IP2666 GFP + ) were grown overnight in 2xYT media. The droplets containing established *Yptb* colonies were then exposed to bone marrow-derived macrophages (BMDMs) collected from C57/BL6 mice. Droplets were then fixed with 4% PFA before visualization by brightfield and fluorescence microscopy.

### Double-layered agarose droplets

The initial agarose droplets were generated using a 40 × 40 μm device as described in the “Droplet Production” method of this paper. Before the oil was removed with 16% PFO, droplets were incubated at 4 °C for 12 min with occasional shaking. Oil-free droplets were added to 37 °C pre-warmed media containing dissolved 1% Seaplaque agarose and then well vortexed. This mixture was then encapsulated into droplets using an 80 μm wide × 40 μm high device, which were designed without filters for the aqueous channel to prevent clogging caused by the initial agarose droplets. Resulting droplets were then gelled at 4 °C for 12 min before culture and visualization by microscopy.

### Fluorescence-activated cell sorting of agarose droplets

After cell growth, the agarose droplets were incubated at 4 °C for 20 min with occasional shaking to ensure that droplets were gelled and stable enough for fluorescence-activated cell sorting (FACS). In total, 16% PFO was used to remove oil as described above, and then agarose droplets were washed several times in 1 ml of PBS by centrifugation at 500 × *g*. Immediately before running the samples on the flow cytometer, agarose droplets were thoroughly vortexed to reduce the clumping of droplets which would otherwise result in clogging of the flow cytometer nozzle (85 um). Acquisition was done using BD FACSAria II (Becton Dickinson, San Jose, CA), and the data were analyzed with Flow Jo software (Becton Dickinson, San Jose, CA).

### Statistical analysis

All statistical analyses were performed using GraphPad Prism version 8.1.2 Mac OS X (GraphPad Software, San Diego, California USA, www.graphpad.com). The specific statistical analyses used can be found in the figure legends. Data was tested for normality with a Kolmogorov–Smirnov test followed by an appropriate (non)parametric test as indicated. All error bars displayed are standard error of the mean.

### Reporting summary

Further information on research design is available in the Nature Research Reporting Summary linked to this article.

## Supplementary information

Supplementary Information

Supplementary Data 1

Supplementary Data 2

Supplementary Data 3

Supplementary Data 4

Supplementary Data 5

Supplementary Data 6

Reporting Summary

Description of Additional Supplementary Files

## Data Availability

All data and genetic material used for this paper are available from the authors on request. All sequence data can be found under the NCBI Sequence Read Archive accession SRP154922. Source data for Figs. 2, 3, 5, 6, 7, and Supplementary Figs. 2 and 3 are available in the Source Data file.

## Source data

Source Data

## References

[1] Langridge GC (2009). Simultaneous assay of every Salmonella Typhi gene using one million transposon mutants. Genome Res..

[2] Gawronski JD, Wong SMS, Giannoukos G, Ward DV, Akerley B (2009). Tracking insertion mutants within libraries by deep sequencing and a genome-wide screen for Haemophilus genes required in the lung.. J. Proc. Natl Acad. Sci. USA.

[3] Goodman AL (2009). Identifying genetic determinants needed to establish a human gut symbiont in its habitat. Cell Host Microbe.

[4] van Opijnen T, Bodi KL, Camilli A (2009). Tn-seq: high-throughput parallel sequencing for fitness and genetic interaction studies in microorganisms. Nat. Methods.

[5] Bronner IF (2016). Quantitative insertion-site sequencing (QIseq) for high throughput phenotyping of transposon mutants. Genome Res..

[6] Guo Y (2013). Integration profiling of gene function with dense maps of transposon integration. Genetics.

[7] Barquist L, Boinett CJ, Cain AK (2013). Approaches to querying bacterial genomes with transposon-insertion sequencing. RNA Biol..

[8] Carter R (2014). Genomic analyses of pneumococci from children with sickle cell disease expose host-specific bacterial adaptations and deficits in current interventions. Cell Host Microbe.

[9] van Opijnen T, Camilli A (2013). Transposon insertion sequencing: a new tool for systems-level analysis of microorganisms. Nat. Rev. Microbiol..

[10] Rowe HM (2019). Bacterial factors required for transmission of Streptococcus pneumoniae in mammalian hosts. Cell Host Microbe.

[11] Veening JW, Smits WK, Kuipers OP (2008). Bistability, epigenetics, and bet-hedging in bacteria. Annu. Rev. Microbiol..

[12] Saether BE, Engen S (2015). The concept of fitness in fluctuating environments. Trends Ecol. Evol..

[13] Cameron DE, Urbach JM, Mekalanos JJ (2008). A defined transposon mutant library and its use in identifying motility genes in Vibrio cholerae. Proc. Natl Acad. Sci. USA.

[14] Fernandez-Martinez LT (2011). A transposon insertion single-gene knockout library and new ordered cosmid library for the model organism Streptomyces coelicolor A3(2). Antonie Van. Leeuwenhoek.

[15] Liberati NT (2006). An ordered, nonredundant library of Pseudomonas aeruginosa strain PA14 transposon insertion mutants. Proc. Natl Acad. Sci. USA.

[16] Lin T (2012). Analysis of an ordered, comprehensive STM mutant library in infectious Borrelia burgdorferi: insights into the genes required for mouse infectivity. PLoS One.

[17] Wetmore KM (2015). Rapid quantification of mutant fitness in diverse bacteria by sequencing randomly bar-coded transposons. mBio.

[18] Shapiro, H. M. *Practical Flow Cytometry* 4th edn. (Wiley-Liss, 2003).

[19] Garcia P, Gonzalez MP, Garcia E, Lopez R, Garcia JL (1999). LytB, a novel pneumococcal murein hydrolase essential for cell separation. Mol. Microbiol..

[20] Domenech A, Slager J, Veening JW (2018). Antibiotic-induced cell chaining triggers Pneumococcal competence by reshaping quorum sensing to autocrine-like signaling. Cell Rep..

[21] Davies DG (1998). The involvement of cell-to-cell signals in the development of a bacterial biofilm. Science.

[22] Boedicker JQ, Vincent ME, Ismagilov RF (2009). Microfluidic confinement of single cells of bacteria in small volumes initiates high-density behavior of quorum sensing and growth and reveals its variability. Angew. Chem. Int. Ed. Engl..

[23] Fournier T, Medjoubi NN, Porquet D (2000). Alpha-1-acid glycoprotein. Biochim. Biophys. Acta.

[24] Lewis AL, Lewis WG (2012). Host sialoglycans and bacterial sialidases: a mucosal perspective. Cell. Microbiol..

[25] King S.J. (2010). Pneumococcal modification of host sugars: a major contributor to colonization of the human airway?. Molecular Oral Microbiology.

[26] Moye ZD, Burne RA, Zeng L (2014). Uptake and metabolism of N-acetylglucosamine and glucosamine by Streptococcus mutans. Appl. Environ. Microbiol.

[27] Pelissier MC (2014). Structural and functional characterization of the Clostridium perfringens N-acetylmannosamine-6-phosphate 2-epimerase essential for the sialic acid salvage pathway. J. Biol. Chem..

[28] Belaaouaj A (1998). Mice lacking neutrophil elastase reveal impaired host defense against gram negative bacterial sepsis. Nat. Med..

[29] Reeves EP (2002). Killing activity of neutrophils is mediated through activation of proteases by K+ flux. Nature.

[30] Standish AJ, Weiser JN (2009). Human neutrophils kill Streptococcus pneumoniae via serine proteases. J. Immunol..

[31] Peschel A (2002). How do bacteria resist human antimicrobial peptides?. Trends Microbiol.

[32] Beiter K (2008). The capsule sensitizes Streptococcus pneumoniae to alpha-defensins human neutrophil proteins 1 to 3. Infect. Immun..

[33] van der Windt D (2012). Nonencapsulated Streptococcus pneumoniae resists extracellular human neutrophil elastase- and cathepsin G-mediated killing. FEMS Immunol. Med. Microbiol..

[34] Lam J, Chan R, Lam K, Costerton JW (1980). Production of mucoid microcolonies by Pseudomonas aeruginosa within infected lungs in cystic fibrosis. Infect. Immun..

[35] Worlitzsch D (2002). Effects of reduced mucus oxygen concentration in airway Pseudomonas infections of cystic fibrosis patients. J. Clin. Invest.

[36] Sriramulu DD, Lunsdorf H, Lam JS, Romling U (2005). Microcolony formation: a novel biofilm model of Pseudomonas aeruginosa for the cystic fibrosis lung. J. Med. Microbiol..

[37] Domenech M, Garcia E, Moscoso M (2012). Biofilm formation in Streptococcus pneumoniae. Micro. Biotechnol..

[38] Casadevall A, Pirofski LA (2000). Host-pathogen interactions: basic concepts of microbial commensalism, colonization, infection, and disease. Infect. Immun..

[39] Freilich S (2011). Competitive and cooperative metabolic interactions in bacterial communities. Nat. Commun..

[40] Giaouris E (2015). Intra- and inter-species interactions within biofilms of important foodborne bacterial pathogens. Front. Microbiol..

[41] Pestova EV, Havarstein LS, Morrison DA (1996). Regulation of competence for genetic transformation in Streptococcus pneumoniae by an auto-induced peptide pheromone and a two-component regulatory system. Mol. Microbiol..

[42] Steinmoen H, Knutsen E, Havarstein LS (2002). Induction of natural competence in Streptococcus pneumoniae triggers lysis and DNA release from a subfraction of the cell population. Proc. Natl Acad. Sci. USA.

[43] Salvadori G, Junges R, Morrison DA, Petersen FC (2019). Competence in Streptococcus pneumoniae and close commensal relatives: mechanisms and implications. Front Cell Infect. Microbiol.

[44] Kjos M (2015). Bright fluorescent Streptococcus pneumoniae for live-cell imaging of host-pathogen interactions. J. Bacteriol..

[45] Moreno-Gamez S (2017). Quorum sensing integrates environmental cues, cell density and cell history to control bacterial competence. Nat. Commun..

[46] Davis KM (2018). All yersinia are not created equal: phenotypic adaptation to distinct niches within mammalian tissues. Front. Cell Infect. Microbiol..

[47] Davis KM, Mohammadi S, Isberg RR (2015). Community behavior and spatial regulation within a bacterial microcolony in deep tissue sites serves to protect against host attack. Cell Host Microbe.

[48] Schneider CA, Rasband WS, Eliceiri KW (2012). NIH Image to ImageJ: 25 years of image analysis. Nat. Methods.

[49] Kjos M, Veening JW (2014). Tracking of chromosome dynamics in live Streptococcus pneumoniae reveals that transcription promotes chromosome segregation. Mol. Microbiol..

[50] Carpenter AE (2006). CellProfiler: image analysis software for identifying and quantifying cell phenotypes. Genome Biol..

[51] van Opijnen T, Camilli A (2012). A fine scale phenotype-genotype virulence map of a bacterial pathogen. Genome Res.

[52] van Opijnen Tim, Lazinski David W., Camilli Andrew (2015). Genome-Wide Fitness and Genetic Interactions Determined by Tn-seq, a High-Throughput Massively Parallel Sequencing Method for Microorganisms. Current Protocols in Microbiology.

[53] McCoy KM, Antonio ML, van Opijnen T (2017). MAGenTA: a Galaxy implemented tool for complete Tn-Seq analysis and data visualization. Bioinformatics.

[54] Anthony, J. S. & van Opijnen, T. A DAG computation server for fully integrated and automated massively parallel sequencing analyses. https://github.com/jsa-aerial/aerobio (2019).

[55] van Opijnen T, Boerlijst MC, Berkhout B (2006). Effects of random mutations in the human immunodeficiency virus type 1 transcriptional promoter on viral fitness in different host cell environments. J. Virol..

[56] Satyanarayan A, Moritz D, Wongsuphasawat K, Heer J (2017). Vega-Lite: a grammar of interactive graphics. IEEE Trans. Vis. Comput. Graph..

[57] Iyer R, Baliga NS, Camilli A (2005). Catabolite control protein A (CcpA) contributes to virulence and regulation of sugar metabolism in Streptococcus pneumoniae. J. Bacteriol..

[58] Untergasser A (2012). Primer3-new capabilities and interfaces. Nucleic Acids Res..

[59] Koressaar T, Remm M (2007). Enhancements and modifications of primer design program Primer3. Bioinformatics.

