# Supplementary material for: Droplet Tn-Seq combines microfluidics with Tn-Seq for identifying complex single-cell phenotypes
Source: Nat Commun. 2019 Dec 16;10:5729. doi: 10.1038/s41467-019-13719-9 (PMC6914776; doi:10.1038/s41467-019-13719-9)
Supplement: Supplementary file 1 — Supplementary Information [file 41467_2019_13719_MOESM1_ESM.pdf]

**droplet Tn-Seq combines microfluidics with Tn-Seq identifying complex single-cell phenotypes**

Thibault et al.,

**Supplementary Figures 1-4**

**droplet Tn-Seq combines microfluidics with Tn-Seq identifying complex single-cell phenotypes**

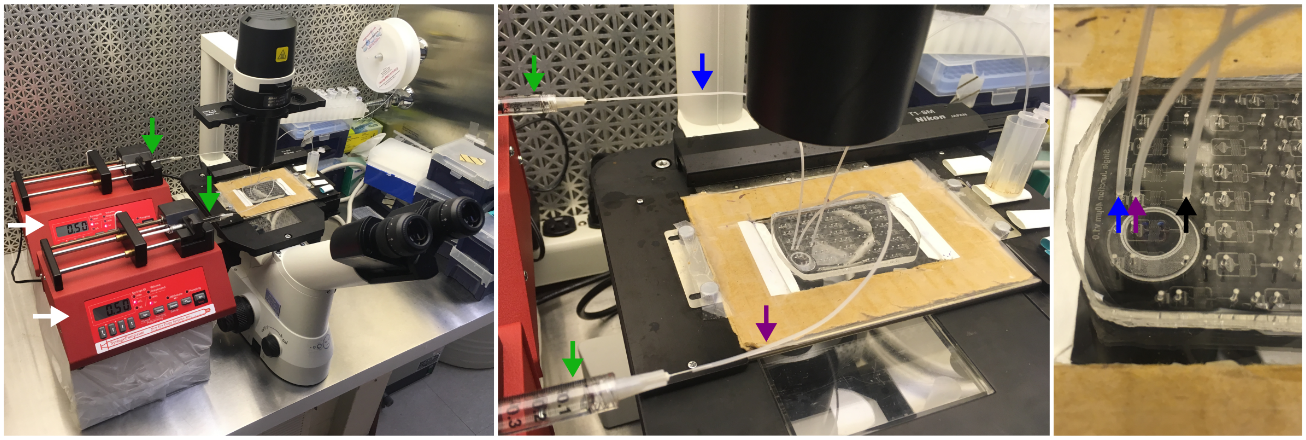


**Supplementary Fig. 1** dTn-Seq droplet microfluidic set-up used to encapsulate bacterial cultures into medium-in-oil droplets. Two pumps (white arrows) control the flow rates from two syringes (green arrows) containing either cell culture or surfactant containing fluorinated oil. Tubing delivers cell culture (purple arrow) and oil (blue arrow) from the syringes to a single device on the microfluidic chip. A third piece of tubing delivers stabilized droplets to a collection tube (black arrow). This system can be used at room temperature to make liquid droplets or be placed in a 37°C warm room to make 1% agarose droplets.

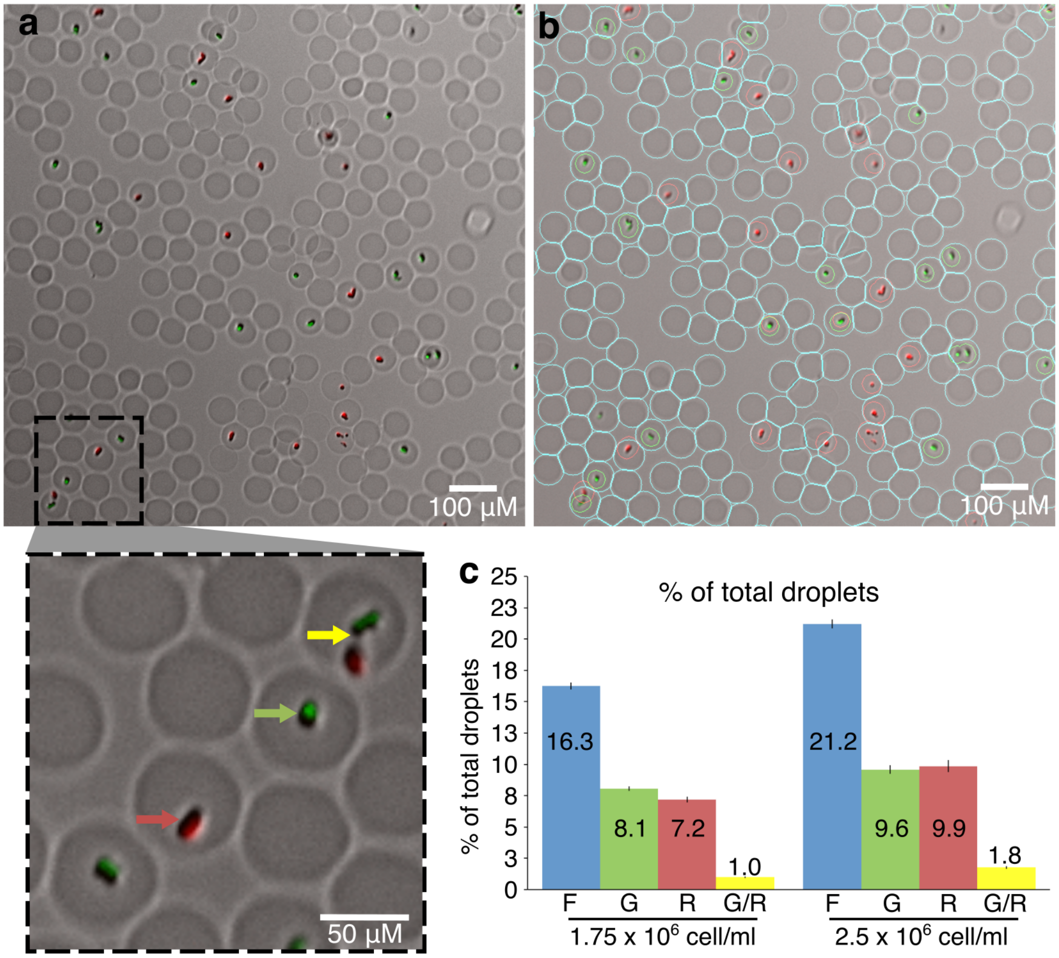


**Supplementary Fig. 2** Cell encapsulation frequencies. Frequencies were determined by first mixing GFP (JWV500) and RFP (MK119) fluorescing *Streptococcus pneumoniae* strains in an equal ratio, encapsulating and growing in agarose droplets, and finally brightfield and fluorescence microscopy was used to determine encapsulation frequencies. **a** CellProfiler software was used to automatically count total droplets, GFP-only (green arrow), RFP-only (red arrow), and droplets containing both GFP and RFP fluorescing cells (yellow arrow) shown in the magnified area. **b** Droplets were identified by using a Hough transform and are outlined in cyan. Red or green microcolonies were identified by thresholding fluorescence images of those respective channels and are highlighted in red or green outlines. **c** The range of cell concentrations used in the study for droplet encapsulation, $1.75\times10^6$ cell/ml - $2.5\times10^6$ cell/ml, respectively yielded 16.3% and 21.2% of the total droplets filled (blue bars) with 1.0-

1.8% of droplets containing multiple cells (yellow bars). F = cell-filled droplet, G = green-cell filled droplet, R = red-cell filled droplet, G/R = droplet with both green and red cells. Measurements are an average of 7 images with at least 10,000 total droplets counted for each of the two cell concentrations. Error bars are standard error of the mean.

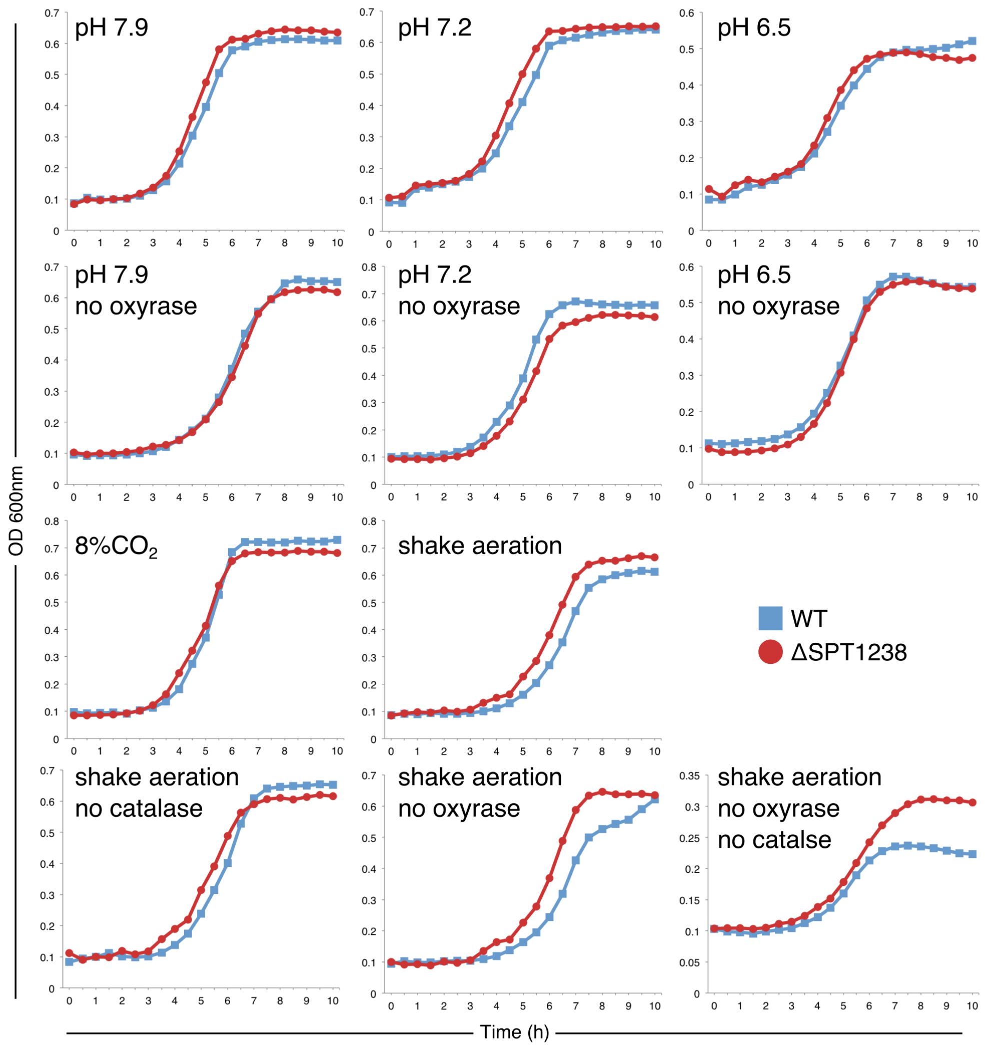


**Supplementary Fig. 3** Fitness of Δ*lytB* is not affected by pH, $CO_2$, $O_2$ or hydrogen peroxide ($H_2O_2$). Each experiment was performed at least three times and shown are representative growth curves.

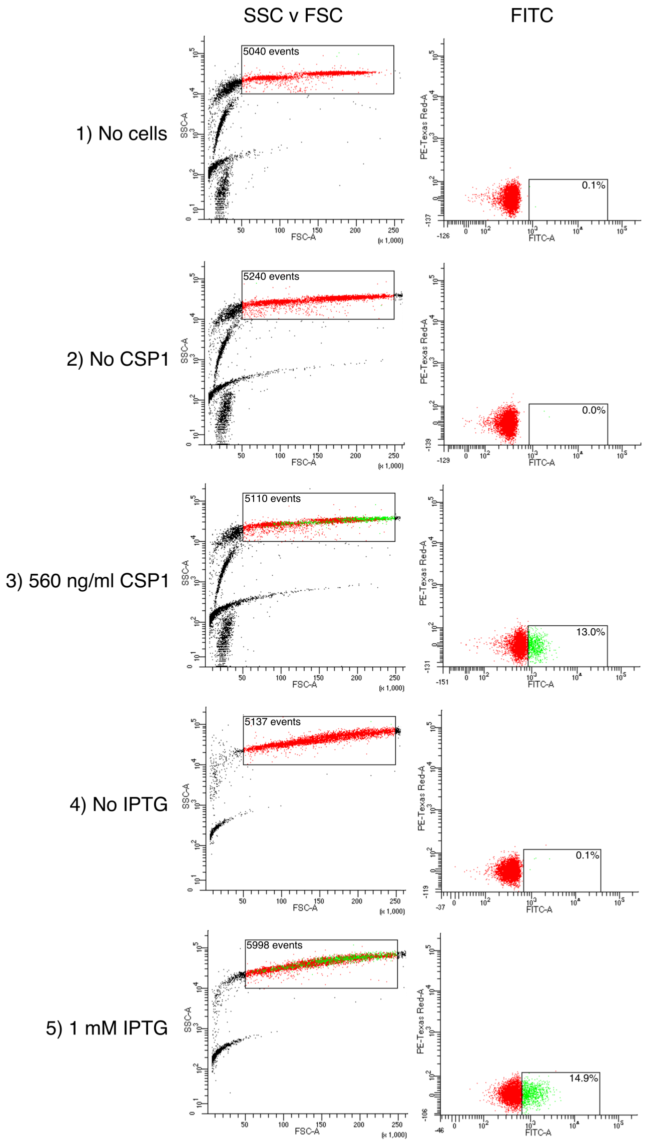


**Supplementary Fig. 4** FACS results from analysis of agarose droplets loaded with a GFP-inducible strain are close to the predicted results based on the initial loading concentration. The samples processed were droplets with no cells (1) or a 40:1 mixture of an IPTG-inducible-CSP strain (ADP112) and a CSP-reporter strain (sfCSPr) respectively (2-5). Agarose droplets were loaded with a final concentration of ~$7\times10^7$ cells/ml of a mixture of ADP112 (~$6.8\times10^7$ cells/ml) and sfCSPr (~$1.75\times10^6$ cells/ml), oil removed, and were treated in the absence or presence of either 560 ng/ml CSP1 or 1mM IPTG for 4hrs. Approximately 5000-6000 events were selected from side scatter (SSC) and forward scatter (FSC) for FACS analysis. While no GFP-positive (FITC) events are

observed for untreated cells both CSP1 treated and IPTG treated cells trigger a similar number of GFP-expressing events in droplets.
