# Supplementary material for: Droplet Tn-Seq combines microfluidics with Tn-Seq for identifying complex single-cell phenotypes
Source: Nat Commun. 2019 Dec 16;10:5729. doi: 10.1038/s41467-019-13719-9 (PMC6914776; doi:10.1038/s41467-019-13719-9)
Supplement: Supplementary file 8 — Reporting Summary [file 41467_2019_13719_MOESM8_ESM.pdf]

nature research

Corresponding author(s): Tim van Opijnen
Last updated by author(s): Nov 5, 2019

Reporting Summary

Nature Research wishes to improve the reproducibility of the work that we publish. This form provides structure for consistency and transparency in reporting. For further information on Nature Research policies, see Authors & Referees and the Editorial Policy Checklist.

Statistics

For all statistical analyses, confirm that the following items are present in the figure legend, table legend, main text, or Methods section.

n/a	Confirmed	
☐	☒	The exact sample size (*n*) for each experimental group/condition, given as a discrete number and unit of measurement
☐	☒	A statement on whether measurements were taken from distinct samples or whether the same sample was measured repeatedly
☐	☒	The statistical test(s) used AND whether they are one- or two-sided<br>*Only common tests should be described solely by name; describe more complex techniques in the Methods section.*
☐	☒	A description of all covariates tested
☐	☒	A description of any assumptions or corrections, such as tests of normality and adjustment for multiple comparisons
☐	☒	A full description of the statistical parameters including central tendency (e.g. means) or other basic estimates (e.g. regression coefficient) AND variation (e.g. standard deviation) or associated estimates of uncertainty (e.g. confidence intervals)
☐	☒	For null hypothesis testing, the test statistic (e.g. *F*, *t*, *r*) with confidence intervals, effect sizes, degrees of freedom and *P* value noted<br>*Give P values as exact values whenever suitable.*
☒	☐	For Bayesian analysis, information on the choice of priors and Markov chain Monte Carlo settings
☒	☐	For hierarchical and complex designs, identification of the appropriate level for tests and full reporting of outcomes
☐	☒	Estimates of effect sizes (e.g. Cohen's *d*, Pearson's *r*), indicating how they were calculated

Our web collection on statistics for biologists contains articles on many of the points above.

Software and code

Policy information about availability of computer code

Data collection	Software and code used for data collection include bcl2fastq, Bowtie 2, ImageJ, Primer3, BD FACSAria II, AutoCad 2016 software (AutoDesk).
Data analysis	Software used for data analysis include CellProfiler, Vega-Lite, Flow Jo, GraphPad Prism version 8.1.2. Sequence analysis was performed with a series of in-house scripts as previously described (van Opijnen et al., 2009; McCoy et al., 2017; Anthony and van Opijnen 2019).

For manuscripts utilizing custom algorithms or software that are central to the research but not yet described in published literature, software must be made available to editors/reviewers. We strongly encourage code deposition in a community repository (e.g. GitHub). See the Nature Research guidelines for submitting code & software for further information.

Data

Policy information about availability of data

All manuscripts must include a data availability statement. This statement should provide the following information, where applicable:

- Accession codes, unique identifiers, or web links for publicly available datasets
- A list of figures that have associated raw data
- A description of any restrictions on data availability

The datasets generated during and/or analysed during the current study are available in the SRA (Accession number: SRP154922)

Field-specific reporting

Please select the one below that is the best fit for your research. If you are not sure, read the appropriate sections before making your selection.

☒ Life sciences ☐ Behavioural & social sciences ☐ Ecological, evolutionary & environmental sciences

For a reference copy of the document with all sections, see nature.com/documents/nr-reporting-summary-flat.pdf

Life sciences study design

All studies must disclose on these points even when the disclosure is negative.

Sample size	Sample size represents the number of biological replicates in an experiment in our study. Sample sizes are 6 for Tn-Seq, 3 for qPCR experiments, 3 or more for growth assays, 3 for survival experiments, 7 images with at least 10,000 total droplets for droplet doublet experiment.
Data exclusions	No data were excluded from the analyses.
Replication	Tn-Seq experiments were successfully replicated in 6 biological replicates. Growth assays were repeated at least three times.
Randomization	Randomization was not necessary for experiments and for all validation experiments not more than one variable was modified.
Blinding	Experimenter was blinded to the sample identities during nucleic acids isolation and sequencing library preparation for all Tn-Seq and dTn-Seq experiments.

Reporting for specific materials, systems and methods

We require information from authors about some types of materials, experimental systems and methods used in many studies. Here, indicate whether each material, system or method listed is relevant to your study. If you are not sure if a list item applies to your research, read the appropriate section before selecting a response.

Materials & experimental systems

n/a	Involved in the study	
☒	☐	Antibodies
☒	☐	Eukaryotic cell lines
☒	☐	Palaeontology
☐	☒	Animals and other organisms
☒	☐	Human research participants
☒	☐	Clinical data

Methods

n/a	Involved in the study	
☒	☐	ChIP-seq
☐	☒	Flow cytometry
☒	☐	MRI-based neuroimaging

Animals and other organisms

Policy information about studies involving animals; ARRIVE guidelines recommended for reporting animal research

Laboratory animals	C57/BL6 mice
Wild animals	Study did not involve wild animals.
Field-collected samples	Study did not involve samples collected from the field.
Ethics oversight	Tufts University and Tufts Medical Center IACUC; protocol# B2019-03 Mechanisms of Yersinia pseudotuberculosis Virulence

Note that full information on the approval of the study protocol must also be provided in the manuscript.

Flow Cytometry

Plots

Confirm that:

☒ The axis labels state the marker and fluorochrome used (e.g. CD4-FITC).

☒ The axis scales are clearly visible. Include numbers along axes only for bottom left plot of group (a 'group' is an analysis of identical markers).

☒ All plots are contour plots with outliers or pseudocolor plots.

☒ A numerical value for number of cells or percentage (with statistics) is provided.

Methodology

Sample preparation	Streptococcus pneumoniae cultured to log phase, diluted, encapsulated into liquid agarose droplets, droplets gelled then added to PBS and then processed by FACS.
Instrument	BD FACSAria II (Becton Dickinson, San Jose, CA)
Software	Flow Jo software (Becton Dickinson, San Jose, CA)
Cell population abundance	Approximately 5000-6000 events (i.e. droplets) were selected from side scatter (SSC) and forward scatter (FSC) for FACS analysis.
Gating strategy	Images of gating strategies is provided in Supplementary Figure 4.

☒ Tick this box to confirm that a figure exemplifying the gating strategy is provided in the Supplementary Information.
