# Supplementary material for: Droplet Tn-Seq combines microfluidics with Tn-Seq for identifying complex single-cell phenotypes
Source: Nat Commun. 2019 Dec 16;10:5729. doi: 10.1038/s41467-019-13719-9 (PMC6914776; doi:10.1038/s41467-019-13719-9)
Supplement: Supplementary file 9 — Description of Additional Supplementary Files [file 41467_2019_13719_MOESM9_ESM.pdf]

Title: Supplementary Data 1
Description: CAD Microfluidic device. Description: CAD layout of the microfluidic device used in this study. 3.

Title: Supplementary Data 2
Description: (d)Tn-Seq Fitness of S. pneumoniae 19F in 1% Agarose. Description: (droplet) Tn-Seq data of S. pneumoniae Taiwan-19F grown in 1% agarose droplets. 4.

Title: Supplementary Data 3
Description: (d)Tn-Seq Fitness of S. pneumoniae TIGR4 in AGP. Description: (droplet) Tn-Seq data of S. pneumoniae TIGR4 grown in droplets and liquid media with AGP as the main carbon source. 5.

Title: Supplementary Data 4.
Description: (d)Tn-Seq Fitness of S. pneumoniae TIGR4 in Elastase. Description: (droplet) Tn-Seq data of S. pneumoniae TIGR4 exposed in droplets and liquid media to the protease Elastase. 6.

Title: Supplementary Data 5.
Description: Adapters and primers. Description: All adapters and primers used in this study. 7.

Title: Supplementary Data 6.
Description: Strains and Plasmids. Description: All bacterial strains and plasmids used in this study.
